# Transcriptomic Analysis Uncovers Immunogenic Characteristics of Ferroptosis for Myocardial Infarction and Potential Therapeutic Prediction of Chinese Herbs

**DOI:** 10.1155/2022/4918343

**Published:** 2022-05-25

**Authors:** Feng Jiang, Zhicong Zeng, Xu Zhou, Meiling Tan, Weiwei Zhang, Mingyue Li, Xiaoduo Zhang, Yinzhi Song, Shanyin Wei, Fengxia Lin

**Affiliations:** ^1^Department of Cardiology, Shenzhen Bao'an Traditional Chinese Medicine Hospital, Guangzhou University of Chinese Medicine, Shenzhen 518000, China; ^2^Wenhua Community Health Service Center, Shenzhen Luohu Hospital Group, Shenzhen 518000, China; ^3^Department of Pharmacy, Shenzhen Bao'an Traditional Chinese Medicine Hospital, Guangzhou University of Chinese Medicine, Shenzhen 518000, China

## Abstract

**Background:**

Inflammation and immune response play a key role in myocardial injury and repair after myocardial infarction (MI), while the relevant regulatory mechanisms of immune infiltration in MI have been fully explored. Ferroptosis is an iron-dependent form of regulated cell death characterized by an excessive accumulation of iron and lipid peroxides and involves in the pathogenesis of myocardial infarction. In the present study, by integrating intelligent data acquisition, data mining, network pharmacology, and computer-assisted target fishing, we developed a highly efficient system for screening immunity- and ferroptosis-related biomarkers and immunomodulatory ability of herbal ingredients.

**Results:**

Immune infiltration analysis of GSE97320 showed significant neutrophil infiltration in the myocardial infarction group compared to the healthy group, and 807 differentially expressed genes (DEGs) were obtained (526 up-regulated and 281 downregulated). Among these DEGs, 73 immune-related and 8 ferroptosis-related DEGs were obtained. Further protein-protein interaction network analysis revealed 30 hub genes. The DEGs were enriched in a total of 107 biological processes, of which neutrophil-related biological processes were the most significant, enriched in 31 cellular components such as bead-binding hemoglobin complex, hemoglobin complex, and enriched in 36 functions such as bead-binding hemoglobin complex and hemoglobin complex. The DEGs were also enriched in 21 KEGG pathways such as lipid-atherosclerosis and formation of neutrophil extracellular traps. Further analysis identified Toll-like receptor-4 (TLR4) as the key gene, and based on TLR4, 17 herbal ingredients and 6 herbal medicines were predicted by using HERB and Coremine databases. Further molecular docking analysis showed that TLR4 could bind to salvianolic acid b and stigmasterol. The molecular dynamics analysis revealed that TLR4 could bind to salvianolic acid b, stigmasterol, and resveratrol in the stable phase with the binding between TLR4 and salvianolic acid b being the most stable.

**Conclusions:**

TLR4 is a key gene that is related to ferroptosis and immune cell infiltration. Further analysis revealed that 17 herbal ingredients and 6 herbal medicines were predicted to have potential interactions with TLR4. These predicted herbal ingredients/medicines may act synergistically to protect against myocardial injury after MI through suppressing neutrophil extracellular traps. The protective effects may be associated with immune cell infiltration and ferroptosis.

## 1. Introduction 

Acute myocardial infarction is the leading cause of human death, though the prognosis of acute myocardial infarction has improved significantly with the more widespread use of invasive treatment strategies such as revascularization and percutaneous transluminal coronary intervention (PCI) [1]. Myocardial cell death after acute myocardial infarction is the main cause of subsequent heart failure and death [2]. Myocardial cell death can be classified as apoptosis, necroptosis, autophagy, ferroptosis, pyroptosis, and mitochondria-mediated necrosis. Ferroptosis is an iron-dependent form of regulated cell death characterized by an excessive accumulation of iron and lipid peroxides [3]. Iron chelation therapy and pharmacological blockade of ferroptosis have been shown to significantly attenuate myocardial injury in mice [4]. Previous studies found that ferroptosis occurred mainly during the reperfusion phase rather than the ischemia phase in the rat model with myocardial ischemia/reperfusion [5]. Based on the importance of ferroptosis in myocardial cell death, therapeutic strategies that target ferroptosis are being investigated. For example, radical trapping antioxidants such as liproxstatin-1 have been suggested as potential drugs [6]. In addition, there is an infiltration of immune cells in the infarcted area after myocardial infarction, and excessive or continuous infiltration of these immune cells can aggravate myocardial damage [7]. It has been found that immune cell infiltration is also essential for the recovery of damaged myocardium after myocardial infarction. For example, the recruitment of regulatory T cells in the area of myocardial infarction of the mice can maintain local homeostasis and promote recovery of the infarcted area [8]. When the infiltrating immune cells remove the necrotic myocardial tissue, the anti-inflammatory monocyte subpopulation will dominate locally and the myocardial infarct zone will begin to repair [9]. This repair mechanism is a function of neutrophils promoting macrophage polarization for the repair phenotype [10]. However, excessive activation and prolonged duration of the inflammatory response can lead to more severe ventricular remodeling and heart failure [11]. Therefore, patients may benefit more from the appropriate and timely use of immunosuppressive drugs, while ferroptosis has been confirmed to play an important role in the inflammatory response [12].

In general, multiple mechanisms are often involved in the development of a disease, and the complexity of Chinese herbal medicine demonstrates its potential for multiple therapeutic pathways. The use of Chinese herbal medicine in the treatment of cardiovascular disease is reaching an unprecedented level of acceptance, and many herbal medicines such as *Panax ginseng*, *Ginkgo biloba,* and *Gynostemma pentaphyllum* have been used for many years in the clinical treatment of cardiovascular disease and have been proven to be effective [13]. For example, icariin has been found to protect against myocardial cell damage due to hypoxia and reoxygenation by inhibiting ferroptosis [14]; resveratrol could protect against myocardial ischemia-reperfusion injury via attenuating ferroptosis [15]. Fu et al. demonstrated that ruscogenin alleviated myocardial ischemia-induced ferroptosis through the activation of branched chain amino acid transaminase 1/branched chain amino acid transaminase 2 [16]. Fan et al. demonstrated that baicalin could prevent myocardial ischemia/reperfusion injury through inhibiting cyl-CoA synthetase long-chain family member 4-mediated ferroptosis [17]. Studies also demonstrated that naringenin alleviated myocardial ischemia/reperfusion injury by regulating the nuclear factor-erythroid factor 2-related factor 2/System xc-/glutathione peroxidase 4 (GPX4) axis to inhibit ferroptosis [18]. Recently, Lu et al. showed that britanin relieved ferroptosis-mediated myocardial ischemia/reperfusion damage by upregulating GPX4 [19]. Thus, we predicted herbal medicines for the treatment of acute myocardial infarction from the perspective of immune cell infiltration and ferroptosis. In the present study, by integrating intelligent data acquisition, data mining, network pharmacology, and computer-assisted target fishing, we developed a highly efficient system for screening immunity- and ferroptosis-related biomarkers and immunomodulatory ability of herbal ingredients.

## 2. Methods

### 2.1. Extraction of Immune- and Ferroptosis-Related Differentially Expressed Genes (DEGs)

GSE97320 (https://www.ncbi.nlm.nih.gov/geo/query/acc.cgi?acc=GSE97320) dataset from Gene Expression Omnibus (GEO) database was included in this study for analysis, and the dataset was contributed by Meng F from Department of Cardiology, China-Japan Union Hospital, Jilin University. In the GSE97320 dataset, three patients with acute myocardial infarction and three healthy people in Northeast Chinese Han people were recruited, total RNA of each sample were extracted from peripheral blood to hybridize with Affymetrix microarrays. Immune cell infiltration analysis was performed by using the cibersort R package for simulation 1000 times after normalization of the genetic data set using the limma R package, and the results were filtered based on adjusted *p* value (adj. P). Differentially expressed genes (DEGs) (log2FC ≥ 1, adj. *P* < 0.05) were also screened using the limma R package in R software. In addition to immune cells, we also downloaded immune-related genes from the ImmPort database (https://www.immport.org/home), and the immune-related genes were intersected with DEGs to obtain immune-related DEGs. The ferroptosis gene set was downloaded from the FerrDb database (http://www.zhounan.org/ferrdb/), and the ferroptosis-related genes were then intersected with DEGs to obtain the ferroptosis-related DEGs. The workflow of the analysis is summarized in Figure S1. The databases used in the present study are summarized in Table 1.

### 2.2. Screening of Hub Genes

The DEGs were imported into the String database for protein−protein interaction (PPI) network analysis with the medium confidence ≥0.400 and PPI enrichment *p* value <1.0e−16. Active interaction sources including text mining, experiments, databases, co-expression, neighborhood, gene fusion, and co-occurrence were applied. The constructed PPI network was imported into Cytoscape for visualization and screening of the top 30 scoring hub genes using the cytohubba plugin (Degree algorithm).

### 2.3. Gene Ontology (GO) and Kyoto Encyclopedia of Genes and Genomes (KEGG) Enrichment Analyses of DEGs

GO and KEGG enrichment analyses of DEGs were performed by using the clusterProfiler R package (pvaluecutoff = 0.05, qvaluecutoff = 0.05) and the top 20 pathways were selected for visual presentation. Subsequently, up- and downregulated genes enriched on immune-related KEGG pathways were tagged using the Color tool of the KEGG database (https://www.kegg.jp/kegg/mapper/color.htmlv).

### 2.4. The Key Genes and Predicted Herbs

The hub genes from DEGs, ferroptosis-related DEGs, and immune-relate DEGs genes were intersected to obtain the original key genes. We considered that the significance of the role of these original key genes in ferroptosis and immune infiltration after myocardial infarction may be different, thus, we also matched these genes with genes in the immune-related KEGG pathway to obtain key genes. Based on key genes, the Herb (http://herb.ac.cn/) and Coremine databases (https://coremine.com/medical/#search) were used to predict the target herbs and herbal ingredients.

### 2.5. Molecular Docking and Molecular Dynamics Analysis of Key Genes to Active Herbal Ingredients

The 2D structure of herbal ingredients was downloaded from the PubChem database (https://pubchem.ncbi.nlm.nih.gov) and was converted to the 3D structure by using Chemoffice (Chem3D 19.0). For the Toll-like receptor (TLR4), to obtain the exact structure, we firstly obtained its identity (ID) in the Uniprot database (https://www.uniprot.org/) and then searched the PDB database (https://www.rcsb.org/) for this ID and selected the high-resolution structure for download. Autodock Vina was used to molecularly dock TLR4 to the active ingredients of Chinese herbal medicines, and to verify whether the predicted Chinese herbal medicines could bind key genes for efficacy. The model with the lowest binding free energy was selected as the best model. The molecular dynamics analysis was then performed using Gromacs software (http://www.gromacs.org/) for the receptor and ligand docking models (30 ns), as follows: the simulated systems were all solventized in a dodecahedron box with an accompanying periodic boundary condition of 1 ns, and the system was first stabilized in equilibrium at PVT of 500 ps and 1000 ps, followed by the Nose-Hoover heat bath coupling algorithm and Parrinello-Rahman pressure coupling method were used to bring the system temperature and pressure to 300 k and 1 bar, respectively, and finally the molecular dynamics of the complex was simulated for 30 ns. The simulation parameters are: non-bonded interaction cutoff = 1.2 nm, particle mesh Ewald calculation of long-range electrostatic interaction with a Coulomb radius of 1.2 nm and a time step of 2 fs, temperature coupling using modified Bredesen with a target temperature of 300 k for both the complex and water and a coupling time constant of 0.1 ps, pressure coupling using the Parrinello–Rahman algorithm with a target pressure of 1 bar and a coupling time constant of 2 ps; root-mean-square deviation (RMSD) was used as the evaluation index for the stability of the complexes, and the smaller the average RMSD, the more stable the binding.

## 3. Results

### 3.1. Collection of Immune- and Ferroptosis-Related DEGs and PPI Network Alayis of DEGs

**Figure 1:**
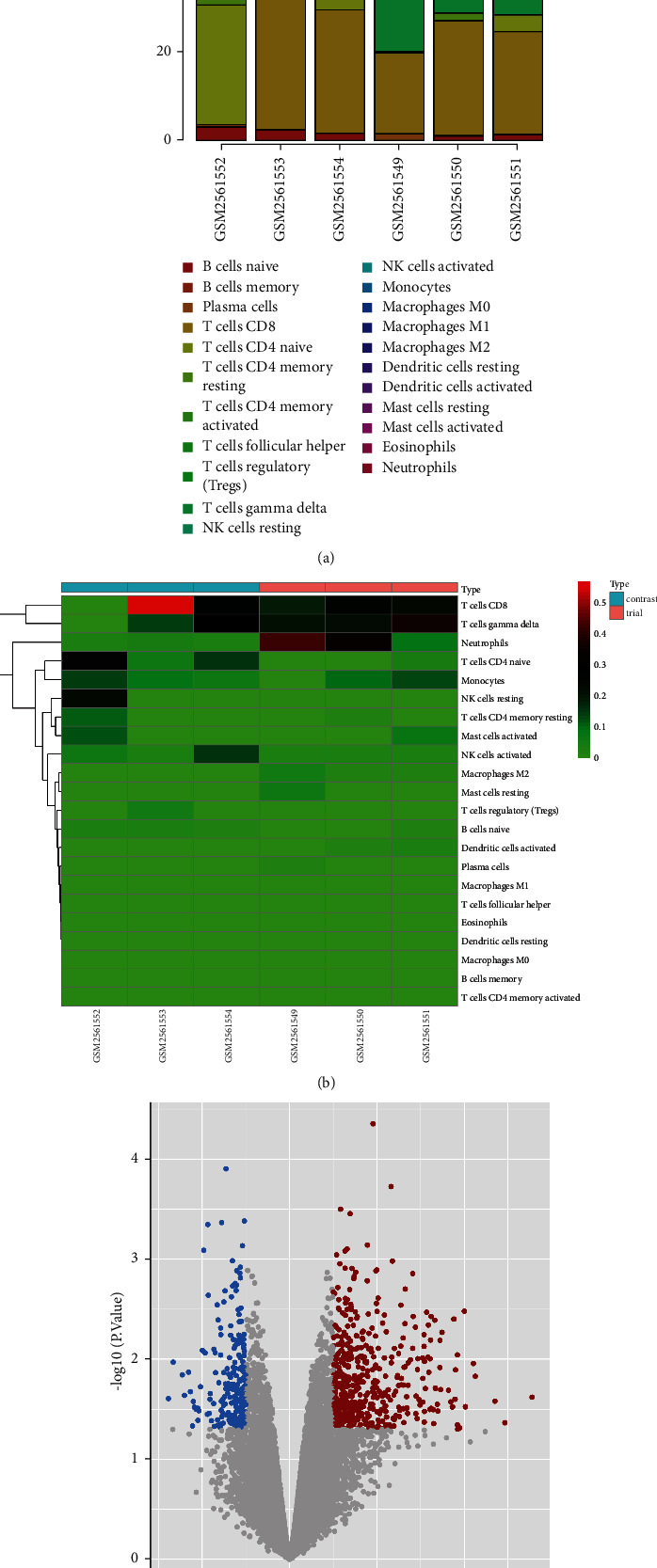
Analysis of DEGs. (a) Percentage of cell types in the individual sample. GSM1561549, GSM1561550, GSM1561551 are the acute myocardial infarction group and GSM1561552, GSM1561553, GSM1561554 are the healthy group. The small rectangles of different colors in each bar represent one type of immune cell and the area represents the level of infiltration. (b) Heatmap of the culturing cells in different groups. The colors in the graph range from green to red, representing higher levels of infiltration, with each row representing one type of immune cell and each column representing one sample. The trial group (pink color) for patients with acute myocardial infarction including GSM1561549, GSM1561550, GSM1561551, while the contrast group (light blue color) for healthy people including GSM1561552, GSM1561553, GSM1561554. Red color indicates up-regulation and green color indicates downregulation. (c) Volcano plot of the DEGs between myocardial infarction group and normal control group. Genes with log2FC ≥ 1 and adj *P* < 0.05 in red are marked as up-regulated genes and genes with −log2FC ≤ −1 and adj *P* < 0.05 in blue are marked as downregulated genes.

**Figure 2:**
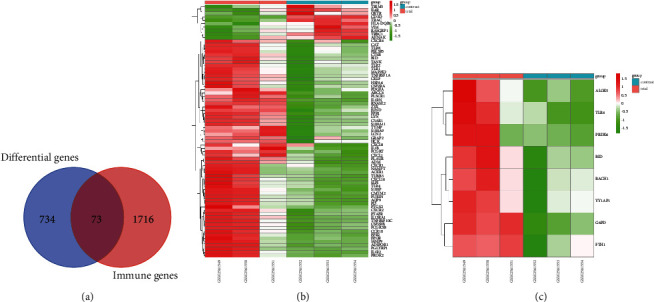
Analysis of immune-related DEGs and ferroptosis-related DEGs. (a) Venn diagram of the overlapped genes. The intersecting part represented 73 immune-related DEGs. (b) Heatmap of the immune-related DEGs. Each line represents an immune-related DEG, and the trial group (pink color) for patients with acute myocardial infarction including GSM1561549, GSM1561550, GSM1561551, while the contrast group (light blue color) for healthy people including GSM1561552, GSM1561553, GSM1561554. Red color indicates up-regulation and green color indicates downregulation. (c) Heatmap of the ferroptosis-related DEGs. Each line represents a ferroptosis-related DEGs, and the trial group (pink color) for patients with acute myocardial infarction including GSM1561549, GSM1561550, GSM1561551, while the contrast group (light blue color) for healthy people including GSM1561552, GSM1561553, GSM1561554. Red color indicates up-regulation and green color indicates downregulation.

The GSE97320 dataset was obtained from GEO database, which contains a total of 6 samples including 3 patients with acute myocardial infarction and 3 healthy subjects. As shown in Figures 1(a) and 1(b), immune infiltration analysis results showed significant neutrophil infiltration in the myocardial infarction group (trial) compared to the healthy group (contrast). A total of 807 DEGs including 526 up-regulated and 281 downregulated genes were obtained. For a clearer presentation, the volcano plot was also used to visualize the DEGs (Figure 1(c)). For the immune-related genes, we obtained 1789 immune-related genes from the ImmPort database, and 73 immune-related DEGs were acquired by intersecting DEGs (Figure 2(a)). The immune-related DEGs were visualized by the Heatmap plot (Figure 2(b)). Besides, we obtained 173 ferroptosis-related genes from the FerrDb database, and by taking intersections with DEGs, we obtained 8 ferroptosis-related DEGs. The heatmap of the ferroptosis-related DEGs is shown in Figure 2(c).

**Figure 3:**
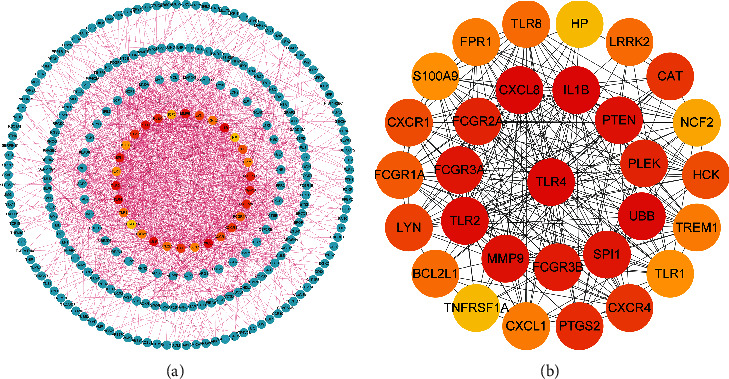
PPI network analysis. (a) PPI network of the DEGs. This figure shows the PPI network, and the centermost circle is the 30 hub genes. (b) PPI network of the 30 hub genes. Each node represents a protein encoded by a gene, and the side lines represent protein-protein interactions, the more side lines, the more important the node. The rank of connection degree is represented by different degrees of color (from red to yellow).

The DEGs were imported into the String database for protein-protein interaction (PPI) network analysis and the PPI network is shown in Figure 3(a). Furthermore, the 30 hub genes from PPT network were screened by Cytohubba (Figure 3(b)).

### 3.2. GO and KEGG Enrichment Analysis of DEGs

**Figure 4:**
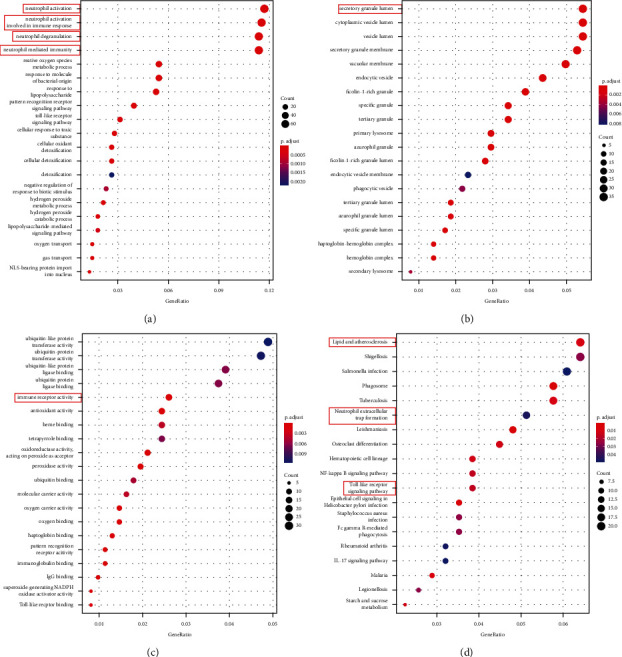
GO and KEGG enrichment analyses. (a) This is a graph of GO biological process enrichment, with increasing significance from blue to red, each bubble represented a biological process and the size of bubble represented the ratio of DEGs enriched in the biological process. (b) This is a graph of GO cellular component enrichment, each bubble represented a cellular component with increasing significance from blue to red, and the size of bubble represented the ratio of DEGs enriched in the cellular component. (c) This is a graph of GO molecular function enrichment, each bubble represented a molecular function with increasing significance from blue to red, and the size of bubble represented the ratio of DEGs enriched in the molecular component. (d) This is a graph of KEGG enrichment, each bubble represented a KEGG pathway with increasing significance from blue to red, and the size of bubble represented the ratio of DEGs enriched in the pathway.

**Figure 5:**
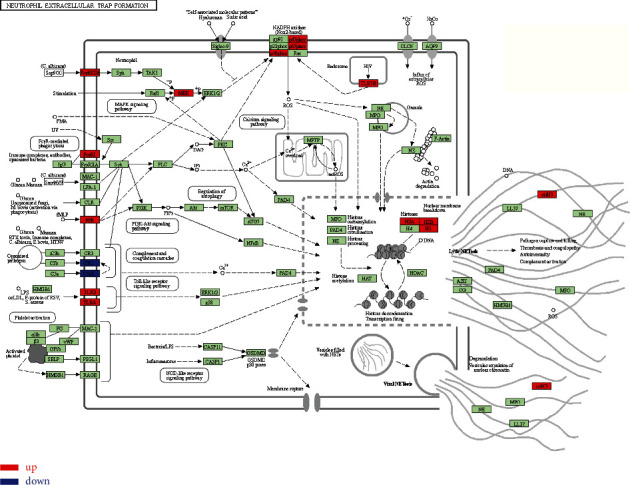
KEGG pathway visualization. This map shows the neutrophil trap pathway, and the DEGs were marked red for up-regulation and blue for downregulation in this pathway.

The DEGs derived from GSE97320 dataset were further processed for GO and KEGG enrichment analysis. In the GO enrichment analysis, a total of 107 GO terms such as neutrophil activation and neutrophil-mediated immunity were enriched in biological processed category (Figure 4(a)). A total of 31 GO terms such as secretory granule lumen were enriched in the cellular component category (Figure 4(b)). A total of 36 GO terms such as immunoreceptor activity and antioxidant activity were enriched in the molecular function category (Figure 4(c)). In addition, we also performed the KEGG enrichment analysis, and we obtained a total of 19 pathways such as neutrophil extracellular trap formation, lipid, and atherosclerosis, and toll-like receptor signaling pathway. As neutrophil extracellular trap formation pathway is associated with leukocytes, we tagged DEGs enriched in this pathway and selected them for further study. The top 20 enriched pathways are shown in Figure 4(d), and the neutrophil extracellular trap formation pathway is illustrated in Figure 5.

### 3.3. The Key Genes and Targeted Herbs

In this study, we found an interesting gene, TLR4, which was presented in immune-related DEGs, ferroptosis-related DEGs, hub genes, and DEGs enriched immune-related KEGG pathway-neutrophil extracellular trap formation, thus, we considered it as the key gene. Based on TLR4, we directly predicted 17 herbal ingredients such as salvianolic acid b, stigmasterol and resveratrol, and 6 herbal medicines such as Radix Salviae liguliobae, Rhizoma Sparganii, and Rhizoma Coptidis in HERB and coremine databases. We found that the 17 herbal ingredients can be extendedly matched to 467 herbal medicines through the HERB database. However, since all data in the Herb database were derived from high-throughput experiments and there is a lack of solid evidence for the extended predicted herbal medicines, thus, we only showed 17 ingredients and 6 herbal medicines in this study (Table 2).

### 3.4. Molecular Docking and Molecular Dynamics of Key Genes with Herbal Ingredients

**Figure 6:**
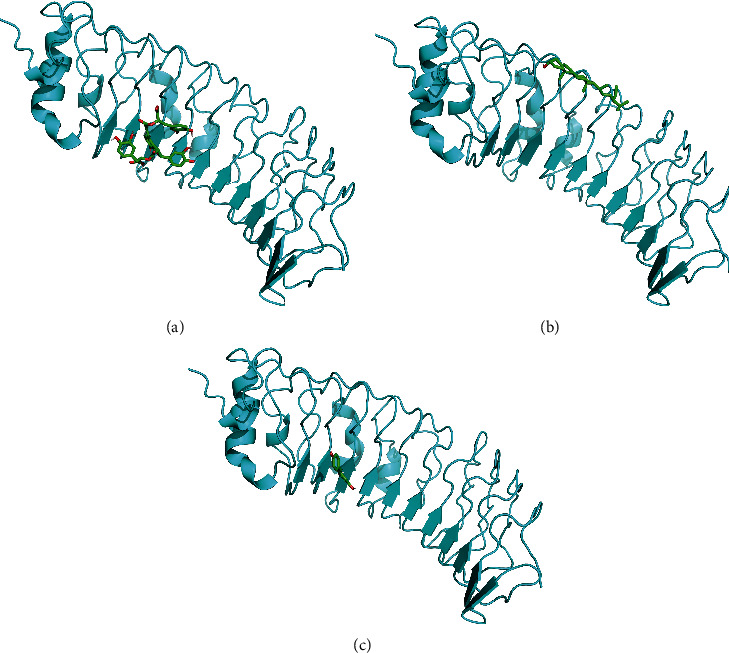
Molecular docking between TLR4 and respective compounds. (a) The best model of molecular docking of salvianolic acid b to TLR4 with a minimum binding free energy of −7.6 kcal/mol. (b) The best model of binding of stigmasterol to TLR4 with a minimum binding free energy of −6.7 kcal/mol. (c) The best model of binding of resveratrol to TLR4 with a minimum binding free energy of −6.2 kcal/mol.

**Figure 7:**
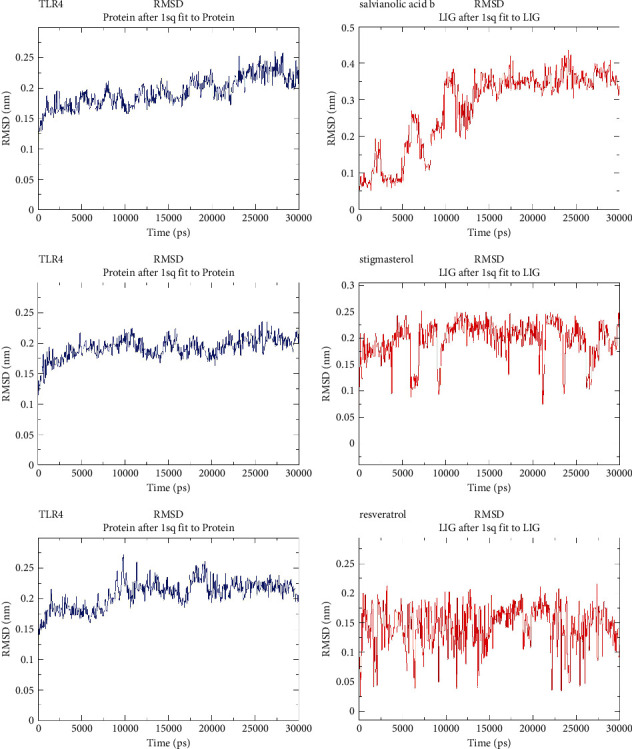
Molecular dynamic analysis between TLR4 and respective compounds.

We found that two of the 17 directly predicted herbal components and 6 herbs corresponded to each other, namely, salvianolic acid b-Radix Salviae liguliobae and stigmasterol-Rhizoma Sparganii. Therefore, we molecularly docked TLR4 with salvianolic acid b and stigmasterol, respectively. We selected the model with the lowest binding free energy as the best model (Figure 6). In the best model, it is clear that salvianolic acid b could potently bind to TLR4 than stigmasterol. Molecular dynamics analysis revealed low mean RMSD values of three complexes that TLR4 (0.15–0.25 nm) combine to salvianolic acid b (0.3–0.4 nm), stigmasterol (0.1–0.25 nm), and resveratrol (0.05–0.2 nm) in the stable phase with the binding between TLR4 and salvianolic acid b being the most stable (Figure 7).

## 4. Discussion

In this study, we identified a key gene, TLR4, which was presented in immune-related DEGs, ferroptosis-related DEGs, hub genes, and DEGs enriched immune-related KEGG pathway-neutrophil extracellular trap formation. Studies have demonstrated that neutrophils infiltrated the infarcted tissue and mediated tissue damage through the release of matrix-degrading enzymes and reactive oxygen species after myocardial infarction [20]. Neutrophils also can produce neutrophil extracellular traps (NETs) after TLR4 stimulation [21]. During this period, downregulation of glutathione peroxidase 4 downregulation contributes to the ferroptosis in cardiomyocytes [22]. Ferroptosis, an iron-dependent cell death, plays an important role in myocardial cell death following myocardial infarction. There is a strong link between ferroptosis and immune infiltration, and it has been shown that ferroptosis can promote neutrophil recruitment in damaged myocardial tissue through TLR4 signaling pathway [23]. It has been found that macrophage-2-derived exosomal microRNA-148a attenuated myocardial ischemia-reperfusion injury in rats by inhibiting TLR4 [24]. Knockdown of TLR4 significantly reduced iron and lipid peroxidation and decreased ventricular remodeling and myocardial death in the rat heart subjected to ischemia-reperfusion [25].

In the field of traditional Chinese medicine, ginsenoside Rg1 could reduce inflammatory cardiomyocyte apoptosis by inhibiting TLR4 [26]. Immune-related KEGG pathway-NETs formation was found to be significantly associated with prognosis in patients with myocardial infarction [27]. NETs can expand the infiltration of immune cells by inducing the release of cytokines from macrophages, thereby enhancing the inflammatory response [28]. NETs acted as a scaffold to adhere to red blood cells and platelets and promoted fibrin deposition, leading to thrombosis [29]. This is of great importance for reinfarction after myocardial revascularization or PCI. Because the reinfarction rate can be up to 1.8% at 1 month after myocardial infarction, in-stent thrombus re-formation can account for 76.3% of this rate [30]. NETs are found to be presented only in incomplete atherosclerotic plaques, such as ruptures and erosions, including the adjacent tissues surrounding such plaques, but were not presented in intact plaques and their surrounding adjacent tissues [31]. TLR4 was significantly up-regulated in the NETs formation pathway, which is consistent with the findings of previous studies [32].

After we obtained this key gene (TLR4), we have two ways to predict the target herbal medicine: one is to directly match TLR4 in the HERB database and Coremine database to predict herbal medicines and herbal ingredients, and the other is extendedly to predict herbal medicine based on these ingredients. We predicted 6 herbal medicines and 17 herbal ingredients directly; while in the latter, we predicted 467 herbal medicines indirectly. As we all know, the composition of herbal medicines is very complex, and it is difficult for us to grasp whether these indirectly predicted herbal medicines are meaningful for the regulation of TLR4. HERB database is the world's largest and most advanced traditional Chinese medicine database, which can demonstrate the traditional Chinese medicine-ingredients-gene-disease relationship. More importantly, herbal medicines data of HERB database are derived from high-throughput tests with high confidence [33]. In our analysis, the outcomes of predicted herbal ingredients-herbal medicines were salvianolic acid b-Radix *Salviae liguliobae* and stigmasterol-*Rhizoma Sparganii*. Molecular docking of the two herbal ingredients with TLR4 separately revealed that salvianolic acid b bound best to TLR4 (−7.6 kcal/mol). Salvianolic acid b has been shown to protect against myocardial ischemia in rats with perfusion injury [34]. It exerted anti-inflammatory effects and attenuated myocardial ischemia-induced injury by inhibiting the TLR4/NF-*κ*B/NLRP3 signaling pathway [35]. Stigmasterol also exhibited anti-inflammatory and antioxidant effects on cerebrovascular diseases to protect against cerebral ischemia-reperfusion injury [36]. Besides, stigmasterol significantly lowered plasma cholesterol levels in the rats (up to 11%), which is the most important direct risk factor for atherosclerosis [37]. In addition, resveratrol is a well-known antioxidant, which has been shown to reduce inflammation in the heart tissues of rats with myocardial ischemia-reperfusion injury via the TLR4/NF-KB signaling pathway [38], and to protect cardiomyocytes from hypoxia-reoxygenation injury through this pathway [39]. Therefore, we also molecularly docked it to TLR4 and found its best model binding free energy to be −6.2 kcal/mol. Further, we subjected these three sets of docking models to molecular dynamics analysis, and the results also confirmed that TLR4 could stably bind to salvianolic acid b, resveratrol, and stigmasterol.

In this study, we also found that the optimal binding sites for salvianolic acid B and resveratrol were very consistent. TLR4, as one of their common targets, may have competing binding sites and therefore the combination may exhibit reduced efficacy or even serious side effects. This gives us a hint as to whether any of the herbs we predicted should not be used in combination when the best binding site is the same. Nevertheless, our findings were mainly based on the prediction of the online databases, and further experimental assays should be performed to consolidate our findings. In addition, our study was only focused on one dataset, and future studies may explore more datasets to consolidate these findings. The results from the molecular docking and molecular dynamics analysis should be interpreted with caution, as molecular docking and molecular dynamics simulation are the computational simulation of the binding ability of compounds to target proteins, and cannot reach the conclusion of excitation or inhibition.

## 5. Conclusion

TLR4 is a key gene that is related to ferroptosis and immune cell infiltration. Further analysis revealed that 17 herbal ingredients and 6 herbal medicines were predicted to have potential interactions with TLR4. These predicted herbal ingredients/medicines may act synergistically to protect against myocardial injury after MI through suppressing NETs. The protective effects may be associated with immune cell infiltration and ferroptosis.

## Figures and Tables

**Table 1:** All relevant software and websites covered in this study.

Database or soft	Web link or soft version
GEO database	https://www.ncbi.nlm.nih.gov/geo/
R And package	Version: R 4.1.1; package: Limma, clusterprofiler
ImmPort database	https://www.immport.org/home
String database	https://cn.string-db.org/
Cytoscape	Version: Cytoscape_v3.9.0; Plug-in: Degree
FerrDb database	http://www.zhounan.org/ferrdb/
KEGG Mapper-Color	https://www.kegg.jp/kegg/mapper/color.htmlv
HERB database	http://herb.ac.cn/
COREMINE database	https://coremine.com/medical/#search
PubChem database	https://pubchem.ncbi.nlm.nih.gov/
ChemOffice	Chem3D 19.0
Uniprot database	https://www.uniprot.org/
PDB database	https://www.rcsb.org/
Autodock Vina	Autodock Vina 1.1.2
Gromacs	http://www.gromacs.org/

**Table 2:** Predicted herbal ingredients and herbs.

Predicted herbal ingredients	Predicted herbs
Resveratrol	Radix salviae liguliobae
Salvianolic acid b	Rhizoma atractylodis macrocephalae
Oleuropein	Rhizoma sparganii
Shikonin	Rhizoma polygoti
Stigmasterol	Rhizoma coptidis
Nordihydroguaiaretic acid	Codonopsis lanceolata trautv
Cinnamaldehyde	
Dioscin	
Gedunin	
Saikosaponin a	
Curcumin	
Punicalagin	
Pinocembrin	
Tauroursodeoxycholic acid	
Puerarin	
Taxol	
Fisetin	

## Data Availability

All the data in the study are available upon reasonable request from the corresponding authors.

## Supplementary Materials

Supplementary Materials Supplemental Figure S1: flowchart of the analysis in the present study.
