# Supplementary material for: Transcriptomic Analysis Uncovers Immunogenic Characteristics of Ferroptosis for Myocardial Infarction and Potential Therapeutic Prediction of Chinese Herbs
Source: Evid Based Complement Alternat Med. 2022 May 25;2022:4918343. doi: 10.1155/2022/4918343 (PMC9159883; doi:10.1155/2022/4918343)
Supplement: Supplementary Materials — Supplemental Figure S1: flowchart of the analysis in the present study. [file 4918343.f1.pptx]

## Slide 1
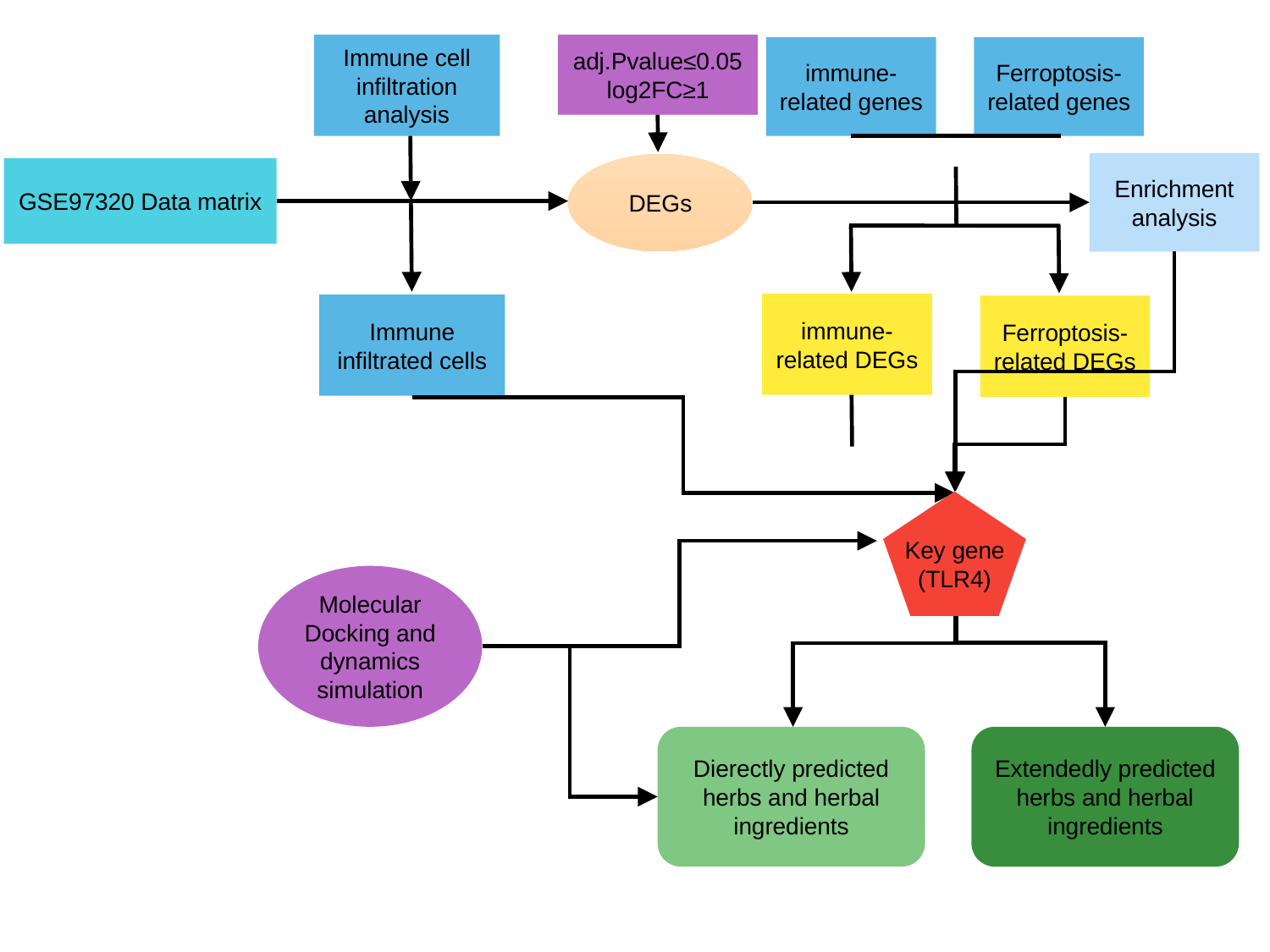

adj.Pvalue≤0.05
log2FC≥1
Immune cell infiltration analysis
immune-related genes
Ferroptosis-related genes
Enrichment analysis
DEGs
GSE97320 Data matrix
immune-related DEGs
Immune infiltrated cells
Ferroptosis-related DEGs
Key gene
(TLR4)
Molecular Docking and dynamics simulation
Dierectly predicted herbs and herbal ingredients
Extendedly predicted herbs and herbal ingredients
